# Reply to “Comments on the Editor Re: The Relationship of Obesity, Nutritional Status and Muscle Wasting in Patients Assessed for Liver Transplantation, Nutrients 2019, 11, 2097.”

**DOI:** 10.3390/nu12030869

**Published:** 2020-03-24

**Authors:** Helen Vidot, Katharine Kline, Robert Cheng, Liam Finegan, Amelia Lin, Elise Kempler, Simone I. Strasser, David Geoffrey Bowen, Geoffrey William McCaughan, Sharon Carey, Margaret Allman-Farinelli, Nicholas Adam Shackel

**Affiliations:** 1 Department Nutrition & Dietetics, Royal Prince Alfred Hospital, Camperdown, NSW 2050, Australia; sharon.carey1@health.nsw.gov.au; 2 Sydney Medical School, The University of Sydney, Sydney, NSW 2006, Australia; katharine.kline@health.nsw.gov.au (K.K.); amelia.lin@health.nsw.gov.au (A.L.); elise.kempler@health.nsw.gov.au (E.K.); simone.strasser@health.nsw.gov.au (S.I.S.); d.bowen@centenary.org.au (D.G.B.); g.mccaughan@centenary.org.au (G.W.M.); 3 Liver Injury and Cancer Centre, Centenary Research Institute, The University of Sydney, Sydney, NSW 2006, Australia; rmcheng@gmail.com; 4 A.W. Morrow Gastroenterology and Liver Centre, Royal Prince Alfred Hospital, Camperdown, NSW 2050, Australia; 5 School of Business, The University of Sydney, Sydney, NSW 2006, Australia; liam.j.finegan@gmail.com; 6 School of Life and Environmental Sciences, Charles Perkins Centre, The University of Sydney, Sydney, NSW 2006, Australia; margaret.allman-farinelli@sydney.edu.au; 7 Medicine, University of NSW, Sydney, NSW 2052, Australia; n.shackel@unsw.edu.au

We thank Drs. Ebadi, Moctezuma-Velazquez, Bhanji and Montano-Loza [1] for their interest and comments on our recent paper [2]. The following is our response to their comments and concerns.

Recent European Society for Parenteral and Enteral Nutrition ESPEN consensus guidelines define sarcopenia as a muscle disease that has a low muscle quantity and quality and is associated with poor physical performance [3]. Due to the retrospective nature of this study, frailty assessments for the cohort were not available and the right psoas muscle area, adjusted for height, was used as an indicator of muscle wasting [4]. However, we would contend that the severe extent of muscle wasting seen in our study is such that it is invariably associated with poor physical performance, and hence, is true sarcopenia [5].

The cut-off levels used in our study were those previously used in a group of colorectal cancer patients to predict major surgical complications, and are likely relevant to patients undergoing assessment for liver transplantation. However, we strongly agree that a liver specific sarcopenia assessment should first be independently validated and then applied in future assessment and management of such patients [5]. More recently, the skeletal muscle index has been used to quantify muscle mass and to identify sarcopenia in patients with end-stage liver disease, using the image analysis software application SliceOmatic (TomoVision, Montreal, Canada) [6]. This method is undoubtedly more accurate than the method employed in our study, but due to limited resources, this application was not available to our group. Consequently, we agree that the reported incidence of sarcopenia may be higher in our study. Furthermore, we agree with Drs. Ebadi, Moctezuma-Velazquez, Bhanji and Montano-Loza that our results further support the weak concordance between sarcopenia and subjective global assessment SGA in patients with cirrhosis, particularly in obese patients with cirrhosis, which has previously been identified [1].

Importantly, malnutrition and sarcopenia are very closely linked, but are also separate processes that are increasingly prevalent in obese individuals with cirrhosis, irrespective of disease aetiology [7]. Individually, these conditions are associated with an increased morbidity and mortality, but when they co-exist, they are associated with higher rates of mortality reduced physical function [7]. Our study highlights the poor sensitivity of the widely used nutritional assessment tool, subjective global assessment (SGA), to detect sarcopenia in patients with cirrhosis [8], particularly in obese cirrhotic patients who are at risk of further muscle wasting and its manifestations [7]. The failure to diagnose sarcopenia in individuals classified as well-nourished using SGA alone potentially results in under-recognition and the progression of sarcopenia [9].

There were significantly lower testosterone levels reported in patients without sarcopenia. However, patients without sarcopenia were predominantly female. As expected, the mean value for testosterone, in male patients, was found to be below the normal range across the cohort, regardless of sarcopenia or BMI. Therefore, as is widely accepted, our study is consistent with low testosterone, having a significant contribution to sarcopenia in male patients [10].

In conclusion, our study emphasizes the limitations of current body composition assessment tools to diagnose sarcopenia in individuals with cirrhosis. It is important that practitioners recognize the increased prevalence of sarcopenia in this group and utilize a multidisciplinary approach to the diagnosis and management of sarcopenia.

## References

[1] Ebadi M., Moctezuma-Velazquez C., Bhanji R.A., Montano-Loza A.J. (2019). Reliable Measures of Sarcopenia in Cirrhosis. Comment on the Relationship of Obesity, Nutritional Status and Muscle Wasting in Patients Assessed for Liver Transplantation, Nutrients 2019,11,2097. Nutrients.

[2] Vidot H., Kline K., Cheng R., Finegan L., Lin A., Kempler E., Strasser S.I., Bowen D.G., McCaughan G.W., Carey S. (2019). The Relationship of Obesity, Nutritional Status and Muscle Wasting in Patients Assessed for Liver Transplantation. Nutrients.

[3] Cruz-Jentoft A.J., Bahat G., Bauer J., Boirie Y., Bruyère O., Cederholm T., Cooper C., Landi F., Rolland Y., Sayer A.A. (2019). Sarcopenia: Revised European consensus on definition and diagnosis. Age Ageing.

[4] Jones K.I., Doleman B., Scott S., Lund J.N., Williams J.P. (2015). Simple psoas cross-sectional area measurement is a quick and easy method to assess sarcopenia and predicts major surgical complications. Colorectal Dis..

[5] Sinclair M. (2019). Controversies in Diagnosing Sarcopenia in Cirrhosis—Moving from Research to Clinical Practice. Nutrients.

[6] Carey E.J., Lai J., Wang C.W., Dasarathy S., Lobach I., Montano-Loza A.J., Dunn M.A. (2017). A Multicenter Study to Define Sarcopenia in Patients with End-Stage Liver Disease. Liver Transplant..

[7] Eslamparast T., Montano-Loza A.J., Raman M., Tandon P. (2018). Sarcopenic obesity in cirrhosis-The confluence of 2 prognostic titans. Liver Int..

[8] Moctezuma-Velazquez C., Ebadi M., Bhanji R.A., Stirnimann G., Tandon P., Montano-Loza A.J. (2019). Limited performance of subjective global assessment compared to computed tomography-determined sarcopenia in predicting adverse clinical outcomes in patients with cirrhosis. Clin. Nutr..

[9] Bunchorntavakul C., Reddy K. (2020). Review article: Malnutrition/sarcopenia and frailty in patients with cirrhosis. Aliment. Pharmacol. Ther..

[10] Moctezuma-Velazquez C., Low G., Mourtzakis M., Ma M., Burak K.W., Tandon P., Montano-Loza A.J. (2018). Association between Low Testosterone Levels and Sarcopenia in Cirrhosis: A Cross-sectional Study. Ann. Hepatol..

